# The Predictors of Complete Pathologic Response in Rectal Cancer during the Total Neoadjuvant Therapy Era: A Systematic Review

**DOI:** 10.3390/cancers15245853

**Published:** 2023-12-15

**Authors:** Emily Flom, Kurt S. Schultz, Haddon J. Pantel, Ira L. Leeds

**Affiliations:** Division of Colon and Rectal Surgery, Department of Surgery, Yale School of Medicine, New Haven, CT 06520, USA

**Keywords:** rectal cancer, pathologic complete response, neoadjuvant therapy, watch-and-wait

## Abstract

**Simple Summary:**

Modern rectal cancer treatment in the form of total neoadjuvant therapy (TNT) offers additional opportunities for organ preservation and consideration for a watch-and-wait (WW) surveillance only approach. Preoperative predictors of pCR after TNT can guide the ideal selection criteria for WW in the current era. An exhaustive literature review found predictors for pCR to include the following: (1) biochemical factors; (2) clinical factors; (3) patient demographics; and (4) treatment sequence for TNT. Additional data from long-term trials using TNT is critical to better inform those considering watch-and-wait approaches following a clinical complete response.

**Abstract:**

The modern rectal cancer treatment paradigm offers additional opportunities for organ preservation, most notably via total neoadjuvant therapy (TNT) and consideration for a watch-and-wait (WW) surveillance-only approach. A major barrier to widespread implementation of a WW approach to rectal cancer is the potential discordance between a clinical complete response (cCR) and a pathologic complete response (pCR). In the pre-TNT era, the identification of predictors of pCR after neoadjuvant therapy had been previously studied. However, the last meta-analysis to assess the summative evidence on this important treatment decision point predates the acceptance and dissemination of TNT strategies. The purpose of this systematic review was to assess preoperative predictors of pCR after TNT to guide the ideal selection criteria for WW in the current era. An exhaustive literature review was performed and the electronic databases Embase, Ovid, MEDLINE, PubMed, and Cochrane were comprehensively searched up to 27 June 2023. Search terms and their combinations included “rectal neoplasms”, “total neoadjuvant therapy”, and “pathologic complete response”. Only studies in English were included. Randomized clinical trials or prospective/retrospective cohort studies of patients with clinical stage 2 or 3 rectal adenocarcinoma who underwent at least 8 weeks of neoadjuvant chemotherapy in addition to chemoradiotherapy with pCR as a measured study outcome were included. In this systematic review, nine studies were reviewed for characteristics positively or negatively associated with pCR or tumor response after TNT. The results were qualitatively grouped into four categories: (1) biochemical factors; (2) clinical factors; (3) patient demographics; and (4) treatment sequence for TNT. The heterogeneity of studies precluded meta-analysis. The level of evidence was low to very low. There is minimal data to support any clinicopathologic factors that either have a negative or positive relationship to pCR and tumor response after TNT. Additional data from long-term trials using TNT is critical to better inform those considering WW approaches following a cCR.

## 1. Introduction

Total neoadjuvant therapy (TNT) for treatment of locally advanced rectal cancer has emerged in recent years as a validated alternative to standard neoadjuvant chemoradiotherapy (CRT) [1,2]. TNT combines multi-agent chemotherapy with chemoradiation therapy with the intent of maximizing presurgical treatment. Recent studies have validated this treatment to be more effective than standard neoadjuvant CRT for disease-free survival and overall survival, as well as for distant micro-metastasis with improved rates of pathologic complete response (pCR) or absence of residual tumor cells (i.e., ypT0N0) [1,2,3,4,5]. Recent NCCN guidelines acknowledge the increasingly prominent role of TNT for rectal cancer, with many phase 2 and phase 3 trials supporting these recommendations [6,7].

In a parallel timeline to the adoption of TNT, watch-and-wait (WW) strategies, or organ-preserving nonoperative management, have also gained attention. Reported in 1998 by Habr-Gama, WW opts for close surveillance for those that achieve a clinical complete response (cCR), or the absence of clinically detectable primary tumor, rather than undergoing proctectomy. Major transabdominal surgery is effectively exchanged for an intensive surveillance paradigm with multiple studies now showing favorable long-term outcomes in select patients [8,9,10,11,12,13,14]. WW avoids the morbidity and mortality of surgery, which includes anastomotic leak, complications related to ostomies, low anterior resection syndrome, and sexual and urinary impairment [15,16,17,18,19,20,21,22]. Current guidelines for watch-and-wait require clinical, radiologic, and endoscopic findings of cCR, including no evidence of residual disease on digital rectal examination, MRI, and endoscopic evaluation [23].

Importantly, the standard of care for rectal cancer therapy is starting to divide between protocols that minimize treatment-related toxicity (i.e., PROSPECT) versus organ preservation approaches that avoid surgery. Thus, identifying those who are most likely to benefit from a WW approach is critical since the trend in treatment approaches for those not undergoing a WW approach is favoring less TNT rather than a universal application. Looking ahead, one’s likelihood of a true pCR after TNT may be the discriminating factor between selecting either of the approaches [24]. Additionally, the higher pCR rates of TNT compared to neoadjuvant CRT alone presents greater opportunities for increased non-operative WW management. A successful watch-and-wait approach relies on a high correlation between cCR and pCR. In the pre-TNT era, the identification of the predictors of pCR after neoadjuvant therapy had been well studied and predictors included tumor size, circumferential extent, the pre-therapeutic T and N clinical stage, tumor fixation, and the distance of the tumor from the anal margin [25,26,27,28,29]. The last meta-analysis to assess the summative evidence on this important treatment decision point predates the acceptance and dissemination of TNT strategies. The purpose of this systematic review was to assess preoperative predictors of pCR after TNT to provide the ideal selection criteria for WW in the current era.

## 2. Materials and Methods

### 2.1. Search Strategy

An exhaustive literature review was performed and the electronic databases Embase, Ovid, MEDLINE, PubMed, and Cochrane were comprehensively searched up to 23 June 2023. Search terms and their combinations included “rectal neoplasms”, “total neoadjuvant therapy”, and “pathologic complete response”. In addition to these database searches, a search by hand for articles on pCR and the response of TNT in rectal cancer was completed based on the references from recent articles. References of included studies as well as other studies from appropriate authors and journals were manually assessed for relevance. The complete search strategy is provided in the Appendix A.

### 2.2. Eligibility Criteria

#### 2.2.1. Inclusion Criteria

Our inclusion criteria were studies involving adults (18 years or older) with clinical stage 2 or 3 rectal adenocarcinoma (T3-4, N0 or TxN1-2M0) who underwent at least 8 weeks (i.e., excluded short course regimens such as the PRODIGE-23 protocol) of neoadjuvant systemic chemotherapy in addition to CRT, also known as TNT. The endpoint reported for treatment was a pathologic complete response status, or pCR, following rectal cancer resection. Due to there being little published data on patient and tumor characteristics leading to pCR after TNT, papers identifying pCR as a secondary endpoint as well as any tumor response to TNT were also included. Only studies in English were included.

#### 2.2.2. Exclusion Criteria

Studies that failed to meet the above criteria were excluded from this systematic review. This included studies of non-adenocarcinoma rectal cancer or those that utilized neoadjuvant CRT only or neoadjuvant chemotherapy only for treatment. Additionally, those with incomplete staging histology of the primary tumor, including local excision or non-total mesorectal excision, were excluded.

### 2.3. Data Extraction

Two authors (EF and KS) reviewed the list of retrieved articles and confirmed eligibility of each study through a comprehensive screen of titles and abstracts. Titles with exclusion criteria were removed. A complete full-text assessment was completed of approved studies (EF and KS). Relevant features of each study were extracted including title, publication year, country of origin, sample size, measure of pCR, and covariates included when comparing those with pCR versus those without pCR. The alternative reviewer then reviewed the extracted data against the full text for accuracy. Duplicate documents were removed. A third reviewer (IL) arbitrated all inter-reviewer discrepancies with resolution through a consensus approach involving all three authors.

### 2.4. Study Quality Assessment

The quality of each article for the primary outcome, pCR, was assessed using the GRADE approach [30]. Each article underwent two-author review and initial quality level assignment (very low, low, moderate, and high) with a third author (EF) serving as arbiter for discrepancies.

### 2.5. Data Synthesis

We required a minimum of 3 studies with similar measures of pCR to perform data synthesis. The primary outcome, pCR, was required for synthesis with the expected common effect measure to be the odds ratio of each covariate being associated with a pCR. Odds ratios were to be combined using a random effects meta-analysis and evaluated with I^2^ for heterogeneity. If not suitable for meta-analysis, a narrative qualitative synthesis alone was planned, which would compare similarities and differences between studies.

### 2.6. Protocol Registration

The protocol was registered with the PROSPERO international prospective register of systematic reviews (CRD42023453571).

### 2.7. Reporting Guidelines

This study was conducted and reported consistent with the PRISMA Preferred Reporting Items for Systematic Reviews and Meta-Analyses framework.

## 3. Results

**Figure 1:**
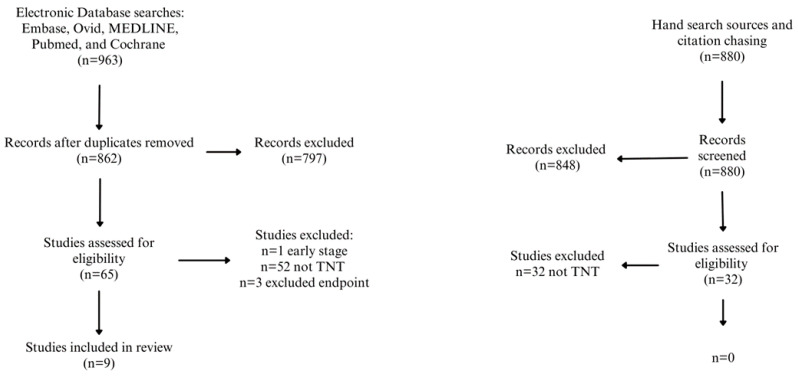
PRISMA flow diagram of systematic review.

A total of 963 related studies were identified from databases Embase, Ovid, MEDLINE, PubMed, and Cochrane. After removing duplicates and those lacking relevant information or endpoints, nine papers met the inclusion criteria for this systematic review (Table 1). See the flow diagram (Figure 1) for an overview of the search process.

### 3.1. An Overview of Study Populations

Studies were published between 2019 and 2023 with patients treated with TNT between 2014 and 2023. TNT regimens included induction chemotherapy (8 cycles of FOLFOX or 5–6 cycles of CAPOX) followed by long-course CRT therapy (25 to 28 radiotherapy fractures with concurrent 5-fluorouracil or oral capecitabine) or short-course CRT therapy (25 Gy in 5 fractions), or long-course CRT followed by consolidation chemotherapy (8 cycles FOLFOX6 or 5–6 cycles CAPOX).

### 3.2. The Benefit of TNT Based on Biochemical Factors

Biochemical predictors, specifically the mutational analysis of tumors and blood markers associated with rectal cancer, were examined in one study to assess predictors to TNT. The retrospective cohort study by Chapman et al. identified 102 patients with stage 2 or 3 rectal adenocarcinoma who underwent TNT, and 38 of those had a complete response (CR) after treatment. A CR was defined as those with a cCR undergoing nonoperative management who remained cancer-free with a median follow-up of 23.3 months or patients who underwent surgery with a pCR. Individuals with a CR were more likely to have a normal pre-treatment CEA level. Patients with a CR were also less likely to have any genetic mutation, which included KRAS, NRAS, BRAF, PIK3CA, p53, APC, FBXW7, or SMAD4 (31.6% vs. 81.6%; *p* < 0.001). Only p53 (21.1% vs. 79%, *p* < 0.05) and SMAD4 (0% vs. 100%, *p* < 0.05) mutations were independently significant in predicting an incomplete response to TNT compared to wild type [31].

### 3.3. The Benefit of TNT Based on Clinical Predictors

Three studies meeting inclusion criteria analyzed laboratory findings for diagnosis and staging, which were collectively grouped as clinical factors that were indicative of a positive or negative response to TNT. While limited data exists on clinical factors best suited for different regimens of TNT, a study by Bedrikovetski et al. introduced personalized TNT treatment based on clinical stage at presentation [32]. Patients at risk of systemic progression (liver or lung metastases, extramural vascular invasion, or abnormal mesorectal or lateral pelvic lymph nodes) received induction chemotherapy whereas patients with a risk of local progression (bulky tumors, T4 extension, or low tumors) received consolidation chemotherapy. A total of 79 patients were included in this study, with 41.8% being treated with an induction TNT approach and 58.2% receiving a consolidation TNT approach. While this study did not compare pCR rates between induction and consolidation chemotherapy or other patient characteristics between the two groups that could affect tumor response, 40.5% of all patients had a cCR and 5.1% of those who had surgery had a pCR. This illustrates how a targeted approach to TNT based on clinical predictors could positively affect tumor response [32].

Another paper by Bedrikovetski et al. prospectively analyzed 118 patients from 2019 to 2022 after TNT for clinical factors which predicted CR after TNT. Treatment was again tailored based on risk of progression, and 30.5% of patients received induction TNT while 69.5% received consolidation TNT. Sarcopenia was diagnosed by universally accepted cut-of points based on CT measurements of the psoas muscle cross-sectional area at the third lumbar vertebral level, normalized for patient height. Univariate and multivariable logistic regression analysis found sarcopenia and hypoalbuminemia to be statistically significant negative predictors of cCR. Sarcopenia was also a negative independent risk factor for overall clinical response (oCR), defined as the proportion of patients who achieved either cCR or pCR [33]. After further analysis, a positive circumferential resection margin was also a negative predictor of oCR [40].

A study from the United States analyzed patient and treatment characteristics associated with pCR for 350 patients in the National Cancer Database that underwent TNT [34]. When stratified by clinical stage, patients with cT4 disease were associated with worse pCR rates and those with cT3 or cN1 disease had improved overall survival [34]. Additionally, patients who underwent TNT had statistically significant improvement in nodal response (ypT + N0) compared to those undergoing neoadjuvant CRT, further leading to improved overall survival for patients with advanced stage cancer [34].

Zhang et al. analyzed the response of clinicopathological characteristics of 120 patients who received TNT with consolidation TNT. Univariate and multivariable analysis found those with MRI staging cN2, tumor diameter ≥ 5 cm before treatment, and lower clearance rate of CEA with elevated CEA levels ≥ 5 ng/mL after CRT being negatively associated with tumor response to TNT [41]. This study highlights the value of selection when evaluating patients for the likelihood of complete response to TNT.

### 3.4. The Benefit of TNT Based on Demographics

As mentioned previously, Chapman et al. found that individuals with a CR were more likely to be younger (median 49 years vs. 55 years in the non-CR group) [31]. Two additional studies assessed the impact of the age of patients on response to TNT, with contradictory results. Foppa et al. assessed the response to both TNT and standard CRT in the treatment of rectal cancer [36]. A total of 16 patients underwent TNT, which included four late-onset patients (age at diagnosis ≥ 50 years) and twelve early-onset patients (age at diagnosis ≤ 49 years). Only 15% of early-onset individuals underwent TNT and the rates of CR stratified by treatment type are not given, limiting the application of this study to assess the response to TNT. While TNT was not a statistically significant risk factor in the multivariable analysis, early-onset disease was a risk factor for an incomplete response to therapy. Of note, a disease-free survival analysis still needs to be completed to assess systemic control for younger patients who underwent TNT [36].

McKenna et al. assessed pCR rates after TNT for young-onset and later-onset patients, with the same age cutoff as the Foppa et al. study [37]. This retrospective review included 72 patients, of which 61% were male with the median age in the young-onset cohort being 43 years and the median age in the later-onset cohort being 64 years. The median follow up was 38 months. Between the younger-onset and later-onset cohorts, there were no statistically significant differences in pCR rates (12% and 15%, respectively), with similar 5-year overall survival rates (86% and 84%, respectively). This contradicts earlier findings which hypothesized early-onset rectal cancer responds differently to TNT [36,37]. Additional data is needed to assess whether age or tumor characteristics associated with early-onset disease are risk factors for poor response to TNT, and therefore, not ideal for WW approaches.

### 3.5. The Benefit of the Order of Therapy Sequence in TNT

In search of a consensus on when and how to administer TNT, namely consolidation compared to induction, for those pursuing non-operative management, two studies analyzed the sequence of chemotherapy and CRT in TNT. The first study was a 2022 multi-institutional study examining CR, again defined as a cCR for non-operative management and pCR in patients who underwent total mesorectal excision, between two types of TNT regimens. This retrospective study by Moyer et al. compared induction chemotherapy with long-course CRT vs. short-course radiation and consolidation chemotherapy. Of the 167 patients, 84 received induction chemotherapy and 83 received consolidative chemotherapy. This study found no difference in cCR between the induction and consolidation groups (49% and 53% respectively, *p* = 0.659), and CR (43% and 53% respectively, *p* = 0.189). A total of 39 (47%) consolidation chemotherapy patients underwent nonoperative management with cCR and 17 (20%) induction chemotherapy patients underwent nonoperative management with cCR. This discrepancy could be because 56% of induction chemotherapy patients did not have a complete re-staging, possibly resulting in more operative management due to an inability to assess for cCR. In this study, there was not a superior TNT sequence for those that wished to achieve organ preservation [38].

Conversely, the Organ Preservation in Patients with Rectal Adenocarcinoma (OPRA) trial, a randomized, multi-institutional, nonblinded phase II trial in the US, found superior outcomes for rectal preservation with consolidation chemotherapy as the TNT regimen. This study randomly assigned 324 patients to either induction chemotherapy or consolidation chemotherapy, with those with a cCR offered WW. Organ preservation, or non-operative management, after cCR was an endpoint along with disease-free survival. The median age of patients was 59 years in the induction group and 56 years in the consolidation group, with a median follow up of 3 years. A multivariable Cox regression analysis found clinical T3, clinical nodal metastasis, and induction chemotherapy were associated with tumor regrowth. Approximately half of the patients randomized to CRT with consolidation chemotherapy achieved cCR, leading to higher three year organ preservation rates compared to patients who received induction chemotherapy with CRT (53% vs. 41% respectively, *p*  =  0.01) [39]. As over 50% of the patients who underwent consolidation chemotherapy achieve rectal preservation, this might represent an optimal regimen for patients hoping for a WW approach [39]. Additional studies are needed to provide optimal treatment strategies to individuals who wish to pursue non-operative management.

#### Quality Assessment Summary

Overall, the quality of the studies was low to very low, with one high-quality study (Table 2). Table 2 reproduces the included studies with evidence provided for GRADE quality domains and a study-specific quality assessment. Eight of nine studies were observational, and all of these also had serious bias risks that further impacted their quality assessment. The OPRA trial was a high-quality study, but pCR was not its primary endpoint.

## 4. Discussion

With several phase III trials recently completed or in process, TNT has been proven as a safe and often superior standard form of treatment in patients with locally advanced rectal cancer, with doubled pCR rates and a reduction in distant metastasis compared to standard neoadjuvant CRT, total mesorectal excision, and optional adjuvant chemotherapy based on surgical pathology [1,3,6,7,42]. However, a lack of standardization in treatment duration, type of radiation therapy and chemotherapy, and the possibility of overtreatment for low-risk patients poses a challenge when attempting to identify prospective patients most appropriate for WW [16,42]. Selecting out high risk patients may reduce the risk of WW salvage surgery or distant metastases with disease recurrence after WW.

In this systematic review, we reviewed nine studies for characteristics positively or negatively associated with pCR after TNT. Key findings of predictors included biochemical predictors, such as genetic mutations, clinical predictors, including sarcopenia, hypoalbuminemia and clinical stage at diagnosis, and patient demographics, including age, as well as the order of therapy sequence in TNT for those that desire non-operative management. The overall quality of evidence was generally low to very low, with only one study having a high equality of evidence. The findings of this systematic review suggest that there are minimal data assessing clinicopathological characteristics and their ability to predict pCR after TNT. Future long-term trials on disease recurrence and overall survival are needed to confirm patient selection for TNT.

### 4.1. Biochemical Predictors

Of the data identified, biochemical predictors that increase tumor susceptibility to TNT include a normal pre-treatment CEA level [31] and an absence of genetic mutations (wild type) [31]. While excluded from our analysis, a study by De Felice et al. highlighted an interesting opportunity for personalized TNT treatment based on biochemical predictors. In this phase 2 single-arm trial, mutated Ras-BRAF was treated with induction chemotherapy in the form of FOLFOXIRI plus bevacizumab, whereas wild-type patients received FOLFOXIRI plus panitumumab/cetuximab. Although these were non-traditional regimens of TNT, 32.1% of patients achieved a complete response with these targeted therapies. While the single-arm design does not allow us to examine biochemical predictors of pCR rates between traditional TNT and alternate regimens, it questions whether other treatment regimens should be considered based on biochemical factors [43]. Unfortunately, induction chemotherapy as used in the De Felice study is not as widely recommended compared to consolidation chemotherapy [7,39], making these findings unclear in their generalizability [43]. Additionally, the small sample size limits the power of both the De Felice and Chapman studies [31,43]. Understanding the pretreatment genetic indicators of rectal cancer can guide TNT treatment to obtain pCR, but further randomized, controlled trials and phase 3 trials are needed. The current quality of evidence for biochemical and genetic markers of pCR after TNT is very low.

### 4.2. Clinical Predictors

Four studies total, with three studies combined for a total of 317 patients, assessed clinical predictors of response to TNT. These studies found that sarcopenia, hypoalbuminemia, a positive circumferential resection margin, stage cN2 and cT4, tumor diameter > 5 cm, and lower clearance rate of CEA with elevated CEA levels ≥ 5 ng/mL after treatment were negatively associated with cCR [33,41]. While these findings align with previous studies of sarcopenia associated with a poor response to neoadjuvant CRT [44], the imaging-based assessment of sarcopenia lacks an evidence-based consensus on standardized thresholds and is limited by inter-rater unreliability [45]. Conversely, tailoring TNT based on clinical staging (risk of local recurrence versus systemic progression) was favorable for overall cCR rates [32]. While Bedrikovetski et al. did not analyze cCR rates between different regimens of TNT and specific clinical predictors of improved responses to TNT have yet to be identified, a precision-guided treatment based on predictors may improve WW outcomes. This is supported by other studies that tailored treatment for patients with rectal cancer, including a study by Cercek et al. While not a TNT regimen, patients with mismatch repair-deficient rectal cancer underwent treatment with anti-PD-1 therapy [46]. A total of 100% of these patients had a cCR after this single agent therapy, suggesting modifying treatment based on tumor characteristics may represent an optimal treatment plan for future patients [32,46]. Two studies found advanced cancer staging, specifically cN2 and cT4, to be negatively associated with pCR after TNT. While the McDermott paper is limited by uncertainties in the accuracy of the database as well as a lack of other prognostic information not recorded [34], this sentiment is supported by the positive tumor response after TNT in early rectal cancer as examined by Habr-Gama [47]. While excluded from our analysis due to clinical stage as well as limitations in the implications and generalizability of this study, they examined the effects of extended CRT with consolidation chemotherapy, or tailored TNT, on patients with cT2N0. In this retrospective cohort study of 123 patients, 85.7% of patients had a cCR in the TNT arm compared to 56.6% having cCR after standard CRT. This high rate of cCR, along with a 1- and 5-year surgery free survival of 82% and 67%, respectively, illustrates a high likelihood of true pCR if surgical pathology was pursued, further supporting tailoring treatment based on clinical stage [47]. For all included studies, however, there was relatively low sample size of patients able to be analyzed, representing a population sample limited in size given the relatively new regimen of TNT, decreasing the power of the studies. Further research is needed to better understand how to tailor TNT regimens to patient and tumor factors. The quality of evidence for clinical predictors of pCR after TNT is very low.

### 4.3. Patient Demographics

The three studies that assessed the response of patients to TNT by age had widely varying results. The quality of the evidence is very low as small sample sizes decreased the power of each study, with less than 100 patients between the Foppa and McKenna studies, which specifically analyzed early-onset rectal cancer responses to TNT. Previous studies analyzing the predictive value of age for pCR after neoadjuvant CRT are also contradictory. Zhang et al. in 2020 found young patients (<40 years old) had lower pCR rates [35]. A 2021 study out of Malaysia further supported those findings, with lower pCR rates after neoadjuvant CRT [48]. However, a 2013 study from Memorial Sloan-Kettering, found similar pCR rates despite early-onset rectal cancer [49]. Other studies have shown age is not associated either positively or negatively with pCR [50]. A hypothesis for future research to explore is if younger patients have other tumor and patient characteristics, including more aggressive features, that are not responsive to treatment [48] or if younger patients are more likely to receive maximum and aggressive treatment [36] with no actual differences in pCR rate based on age.

### 4.4. The Order of Therapy Sequence

Two studies, including a randomized phase II trial, assessed outcomes based on the order of therapy sequence, or induction versus consolidation chemotherapy. While Moyer et al. did not find a superior sequencing of TNT, the OPRA trial supports consolidation chemotherapy for those that wish to pursue WW. Additional trials also support consolidation chemotherapy, including the 2019 German trial CAO/ARO/AIO-12. While not included in our analysis due to the abbreviated length of TNT, this multicenter phase II trial randomized 306 patients to either three cycles of FOLFOX before (induction) or after (consolidation) with pCR as the primary endpoint [51,52]. The first report from 2019 showed that consolidation chemotherapy was associated with a higher pCR (25% vs. 17%) and combined pCR and cCR rates (28% vs. 21%) [51]. Long-term results published in 2022 illustrated no significant difference in toxicity, further promoting consolidation chemotherapy with TNT for those prioritizing organ preservation [52]. This is consistent with the previously mentioned OPRA trial, where 50% of patients who underwent consolidation chemotherapy achieved a sustained cCR and therefore, had higher rates of organ preservation [39]. Despite the variability between the three studies, based on the total study population of over 600 patients between the two trials, consolidation chemotherapy appears to be the superior therapy sequence for individuals undergoing WW. The quality of evidence supporting consolidation therapy as a predictor of pCR is moderate, and therefore, consolidation therapy is our most evidenced conclusion from the existing literature for this systematic review.

### 4.5. Future Directions

As TNT is still a relatively new regimen for rectal cancer, there lacks robust predictors of cCR or pCR. In the pre-TNT era, predictors of pCR after neoadjuvant CRT had only recently been shown. A systematic review from 2016 found a lack of predictors of pCR after neoadjuvant CRT, despite its presence in rectal cancer treatment for over 20 years [53]. However, in the last ten years, predictors of pCR after neoadjuvant therapy have been further studied and recent 2023 papers identified potential biomarkers [54] and clinical factors [55] that determine pCR after neoadjuvant CRT. Other studies have described pCR relative to other predictors including tumor size, circumferential extent, the pre-therapeutic T and N clinical stage, tumor fixation, and the distance of the tumor from the anal margin [25,26,27,28,29]. Additional research should be aimed at validating these clinical, biochemical, and patient predictors of pCR after TNT, rather than CRT alone.

Future directions are ongoing, as varying TNT regimens are currently being tested. A study by Bujko et al. compared TNT with short course radiotherapy and three cycles of FOLFOX compared with standard neoadjuvant CRT [56]. This shortened consolidation chemotherapy and radiotherapy TNT had similar rates of pCR compared to CRT, and long-term results published in 2022 had comparable overall survival rates [57]. Additionally, the NOMINATE trial out of Japan is an ongoing, prospective, multicenter randomized phase 2 trial that will assess pCR or cCR ≥2 years after either TNT with consolidation chemotherapy or TNT with induction chemotherapy [58]. Future studies are needed to designate the optimal duration and regimen of TNT for patients in the context of non-operative management.

### 4.6. Limitations

This study is not without limitations. Neoadjuvant regimens across the world are so heterogeneous that making amalgamated conclusions about the existing published literature in the TNT treatment context is hard. The studies that do exist are not directly comparable and most are low to very low quality.

In addition, the priorities of neoadjuvant therapy are continually evolving. For example, the PROSPECT trial is currently leading a wave of interest focused on reducing neoadjuvant therapy exposure [24]. While these countervailing efforts to TNT impair consensus development, they also highlight the importance of further research into predictors of pCR after TNT. If pCR is unlikely, current trends would typically favor reduced neoadjuvant intensity in favor of surgical resection when feasible. Conversely, the best watch-and-wait outcomes have come through a TNT paradigm. Thus, in an era where TNT itself may be under threat as a standard of care, knowing who may benefit the most from TNT will be essential to keeping this option available for the best selected patients.

## 5. Conclusions

The purpose of this systematic review was to assess preoperative predictors of pathologic complete response after total neoadjuvant therapy to provide the ideal selection criteria for watch-and-wait strategies. Despite small sample sizes with heterogenous TNT regimens, predictors were grouped into four categories. Key findings of predictors were biochemical predictors, such as genetic mutations, clinical predictors, including sarcopenia, hypoalbuminemia, and clinical stage at diagnosis, patient demographics, including age, and the order of therapy sequence of TNT for those that desire non-operative management. By confirming the findings in this systematic review and identifying additional pCR predictors in larger patient populations, a more validated and appropriate patient selection for WW can be determined.

## Figures and Tables

**Table 1:** Characteristics of Included Studies.

Study (Publication Year)	Number of Patients	Study Type	Comparators Assessed	Primary End Point	pCR Rate
Chapman et al. [31] (2023)	102	Retrospective cohort study	Sex, age, BMI, race, tumor characteristics such as tumor distance from anorectal ring, CEA level, and clinical staging characteristics such as T-stage CRM, tumor grade, and genetic characteristics such as MSI status, and genetic mutation	CR (pCR or cCR)	21.6% cCR; 23.8% pCR; 37.3% CR
Bedrikovetski et al. [32] personalized TNT (2023)	79	Prospective observational study	Induction vs. consolidation chemotherapy based on clinical stage	CR (pCR or cCR)	40.5% cCR; 5.1% pCR
Bedrikovetski et al. [33] sarcopenia (2023)	118	Prospective observational study	Induction vs. consolidation chemotherapy, ECOG performance status, stage, BMI, RT dose	CR (pCR or cCR)	40.7% oCR; 36.4% cCR; 9.4% pCR
McDermott et al. [34] (2021)	350	Retrospective cohort study	Age, gender, clinical stage, insurance status, income, comorbidity score	pCR, pCR of primary tumor (ypT0) and pCR of nodes (ypN0)	17.5% pCR 18% ypT0 72% ypN0
Zhang et al. [35] (2022)	120	Retrospective cohort study	Clinicopathological data including clinical TNM staging, mesorectal fascia (MRF) and extramural vascular invasion (EMVI), CEA level,	Tumor regression response	N/A
Foppa et al. [36] (2023)	16	Single-center observational (partially retrospective) and ambidirectional parallel-cohort study	Patient demographics including age, and smoking status. Histopathological characteristics including extramural invasion	Incomplete pathological tumor response	N/A
McKenna et al. [37] (2022)	72	Retrospective cohort study	Age (<50 years and ≥50 years)	pCR or cCR	12% pCR and 15% pCR +cCR early onset; 22% pCR and 30% pCR + cCR late onset
Moyer et al. [38] (2023)	167	Multi-center, retrospective cohort study	Induction chemotherapy with long-course chemoradiation (CRT-TNT) vs. short-course radiation and consolidative chemotherapy (SCRT-TNT)	CR (pCR or cCR)	49% cCR and 43% CR and 22.6% pCR in CRT-TNT; 53% cCR and 53% CR and 6% pCR in SCRT-TNT
Garcia-Aguilar et al. [39] (2022)	324	Prospective, randomized multicenter phase II trial	Induction chemotherapy followed by chemoradiotherapy (INCT-CRT) or chemoradiotherapy followed by consolidation chemotherapy (CRT-CNCT)	Disease-free survival (DFS); pCR; organ preservation	20% pCR for INCT-CRT; 35% for the CRT-CNCT; 53% organ preservation for CRT-CNCT; 41% organ preservation for INCT-CRT

Abbreviations: CR—complete response; pCR—pathologic complete response; cCR—clinical complete response; TNT—total neoadjuvant therapy; CRT—chemoradiation therapy; SCRT—short course radiotherapy; INCT—induction chemotherapy; CNCT—consolidation chemotherapy.

**Table 2:** GRADE Quality Assessment of Included Studies.

Study	Study Type	Detractors (Risk of Bias, Inconsistency, Indirectness, Imprecision)	Protective Factors (Large Effect, Dose Response, Opposing Bias)	Overall Quality of Evidence
Chapman et al. [31]	Retrospective cohort study	*Risk of Bias* The small cohort size limited the ability to perform a multivariable analysis for factors independently associated with a complete response. Population was mainly white. *Indirectness* Complete response was defined as either patients with a clinical complete response undergoing nonoperative management or patients undergoing surgery with a pathological complete response. *Imprecision* Microsatellite instability was not routinely evaluated in patients with proficient mismatch repair, which may impact response rates. Mutational analysis was not routinely performed, and the sample size for genetic mutations may have been too small to detect a significant difference in complete responders and incomplete responders. Only patients with induction chemotherapy were included, which does not fully capture current TNT practices.	*Large Effect* 100% of patients with a complete response had a wild-type SMAD4 mutation compared to 80.6% of patients with an incomplete response. In other words, all patients with a SMAD4 mutation did not have a complete response. *Opposing Bias* Patients who underwent consolidative chemotherapy after radiation were excluded to minimize heterogeneity with respect to the neoadjuvant therapy regimen.	Very Low
Bedrikovetski et al. [32] personalized TNT	Prospective observational study	*Risk of Bias* No modeling was performed. *Inconsistency* Only 73.4% of patients completed the planned number of cycles. Seven patients (8.9%) received other tailored treatment regimens including FOLFIRI, TOMOX, Bevacizumab, Pembrolizumab and Panitumumab. *Indirectness* The primary endpoint was complete response rate defined as the proportion of patients who achieved either a clinical complete response or pathological complete response. *Imprecision* Race and ethnicity data were not collected. Eight patients declined surgery after completing TNT despite having residual clinical disease.		Very Low
Bedrikovetski et al. [33] sarcopenia	Prospective observational study	*Risk of Bias* Sarcopenic patients were more likely to have higher ECOG, have higher T and N stages, have shorter course radiotherapy, be non-compliant with radiotherapy, and receive less radiation. The multivariable analysis did not include important predictors for complete response including circumferential resection margin and pretreatment carcinoembryonic antigen. *Inconsistency* There were two different TNT protocols. *Indirectness* The primary endpoint was clinical complete response or pathological complete response. *Imprecision* Sarcopenia cutoffs should be determined within each specific patient population and body mass index category, but this study used universally accepted cut-off points.	*Opposing Bias* Sarcopenic patients completed more planned cycles of chemotherapy.	Very Low
McDermott et al. [34]	Retrospective cohort study	*Risk of Bias* The study was a secondary analysis of an administrative dataset, which has inherent limitations related to the accuracy of the data, selection bias, and unmeasured confounders. *Inconsistency* Radiation doses, length of radiation, and time from radiation completion to surgery were not consistent among patients in the TNT group. *Imprecision* The administrative dataset used (NCDB) does not have a variable for chemoradiation, so assumptions were made to define this variable.	*Large effect* Clinical T4 disease was a significant negative predictor of pCR (OR = 0.2, *p* = 0.02) *Opposing bias* The study performed propensity-score matching.	Low
Zhang et al. [35]	Retrospective cohort study	*Inconsistency* Surgery included low anterior resection, abdominoperineal resection, or Hartmann’s procedure. *Indirectness* The primary endpoint was not stated. The study’s primary focus was tumor regression response and post-treatment carcinoembryonic antigen clearance and not on pathologic complete response. *Imprecision* Only patients with consolidation chemotherapy were included, which does not fully capture current TNT practices.	*Opposing bias* There were no significant differences in operation time, blood loss, postoperative complications, lymph nodes harvested, or lymphovascular invasion between the good response and poor response groups. Surgery was performed regardless of evidence of complete clinical response.	Very Low
Foppa et al. [36]	Single-center observational (partially retrospective) and ambidirectional parallel-cohort study	*Risk of Bias* Mean body mass index and comorbidities were different between the early-onset rectal cancer and late-onset rectal cancer groups. The model adjusted for body mass index but not for comorbidities. More late-onset rectal cancer patients underwent neoadjuvant chemoradiotherapy, but more early-onset rectal cancer patients received total neoadjuvant therapy (15% vs. 1%). The long time span of the study is a limitation as new approaches have developed. *Indirectness* The primary endpoint was the rate of incomplete response. Patients with DNA mismatch repair mutations were excluded. *Imprecision* The confidence interval for age of onset was 1.02 to 3.16 in multivariable analysis.	*Opposing Bias* The study used a strict definition of early-onset rectal cancer according to age. The study focused on preoperatively treated locally advanced rectal cancer patients, which presented a homogenous population in terms of pre-treatment MRI stage between the early-onset and late-onset rectal cancer groups. The early-onset and late-onset rectal cancer groups were balanced in terms of pathological features. Patients enrolled in the watch-and-wait protocol and operated on for a regrowth during follow-up were excluded from analysis, which made the population more homogenous.	Low
McKenna et al. [37]	Retrospective cohort study	*Indirectness* The abstract does not state the study’s primary endpoint, but it can be assumed to be pathologic complete response. *Imprecision* The study was underpowered to detect a difference in pathologic complete response between the young-onset and later-onset locally advanced rectal cancer groups. The pathologic complete response was 12% in the young-onset group compared to 22% in the late-onset group. This appears to be clinically significant, but it was not statistically significant likely due to the small sample size of the study. *Publication Bias* This was a conference abstract.	*Opposing Bias* There were no significant differences in the baseline characteristics between the young-onset and later-onset locally advanced rectal cancer groups. Only 68% of patients were Caucasian.	Very Low
Moyer et al. [38]	Multi-center, retrospective cohort study	*Risk of Bias* The study had a small sample size. The two arms of the study were conducted at different centers by different surgeons. Patients in the induction chemotherapy and long-course chemoradiation group had a shorter interval from completion of total neoadjuvant therapy to surgery compared to the short-course radiation and consolidative chemotherapy group. There might have been a selection bias as to which patients were chosen for the two different total neoadjuvant therapy protocols. Some patients received total neoadjuvant therapy at a non-study center. More patients in the induction chemotherapy and long-course chemoradiation group were younger and had at least 12 lymph nodes obtained compared to the short-course radiation and consolidative chemotherapy group. *Inconsistency* There was a high rate of near complete and incomplete total mesorectal excision. *Indirectness* The primary endpoint was the rate of severe postoperative complications. The secondary outcome was rates of complete response (clinical or pathological).		Very Low
Garcia-Aguilar et al. [39]	Prospective, randomized multicenter phase II trial	*Risk of Bias* The study was nonblinded. (This is a non-serious risk of bias.) *Indirectness* The primary endpoint was disease-free survival.	*Large Effect* For the intention-to-treat population, there was increased organ preservation rates in the chemoradiation and consolidative chemotherapy group compared to the induction chemotherapy and chemoradiation group (53% vs. 41%; *p* = 0.01).	High

## Data Availability

All data from the systematic literature search is available upon request to the corresponding author.

## Appendix A

Ovid: MEDLINE(R) ALL

1  . Rectal Neoplasms/  45915

2  ((. rectum or rectal) adj3 (cancer or neoplasm or carcinoma*)).mp.   34112

3   1 or 2  56045

4   Neoadjuvant Therapy/ 25890

5   (neo$adjuvant adj2 (therap* or treatment*)).mp.  33544

6   4 or 5  33544

7   (pathologic adj complete adj response).mp.  3055

8   3 and 6 and 7  400

9   limit 8 to humans   358

Embase <1974 to 2023 June 27>updated 6/28/23

Embase <1974 to 2023 June 27>

1  rectum cancer/ or rectum carcinoma/  56989

2  ((rectum or rectal) adj3 (cancer or neoplasm or carcinoma*)).mp.  72923

3  1 or 2 72923

4  Neoadjuvant Therapy/  23097

5  (neo$adjuvant adj2 (therap* or treatment*)).mp.  45032

6  4 or 5 45032

7  (pathologic adj complete adj response).mp.  6970

8  3 and 6 and 7     600

9  limit 8 to human  582

Ovid MEDLINE(R) ALL <1946 to June 27, 2023> (DONE)-NO NEW PAPERS

1  Rectal Neoplasms/ 48055

2  ((rectum or rectal) adj3 (cancer or neoplasm or carcinoma*)).mp.  36570

3  1 or 2 59116

4  (total adj neoadjuvant therapy).mp.  273

5  3 and 4   247

6  limit 5 to humans  194

7  (pathologic adj complete adj response).mp.  3481

8  5 and 7   28

9  limit 8 to humans  21

Cochrane

Date Run: 6/27/2023

Comment:

ID Search Hits

#1 (rectum or rectal) NEAR/3 (cancer or neoplasm or carcinoma*) 4332

#2 neo$adjuvant NEAR/2 (therap* or treatment*) 383

#3 pathologic NEAR complete NEAR response 1001

All 3: 2 papers
